# A Comprehensive Overview of Co_3_O_4_ Nanoparticles: Solution Combustion Synthesis and Potential Applications

**DOI:** 10.3390/nano15120932

**Published:** 2025-06-16

**Authors:** Togzhan T. Mashan, Muhammad Hashami, Nurgul S. Bergeneva, Nurgul N. Nurmukhanbetova, Aigul S. Beisebayeva, Meruyert Nazhipkyzy, Gulnar U. Mamatova, Aigerim G. Zhaxybayeva

**Affiliations:** 1 Department of Chemistry, Faculty of Natural Sciences, L.N. Gumilyov Eurasian National University, Astana 010008, Kazakhstan; mashan_tt@enu.kz (T.T.M.); zhaxybayeva_ag_1@enu.kz (A.G.Z.); 2 Department of Chemical Physics and Material Science, Al-Farabi Kazakh National University, 71 Al-Farabi Avenue, Almaty 050040, Kazakhstan; mg.hashami2010@gmail.com; 3 Department of Chemistry, Faculty of Education, Institute of Higher Education Mirwais Khan Nika, Qalat 4001, Zabul, Afghanistan; 4 Institute of Combustion Problems, 172 Bogenbai Batyr Street, Almaty 050012, Kazakhstan; 5 UNESCO Chair in Sustainable Development, Al-Farabi Kazakh National University, 71 Al-Farabi Avenue, Almaty 050038, Kazakhstan; nurgul.bergeneva@kaznu.kz; 6 Scientific Research Laboratory on Water Quality, Department of Chemistry and Biotechnology of the Pedagogical Institute, Sh. Ualikhanov Kokshetau University, 76 Abai Street, Kokshetau 020000, Kazakhstan; nnurmukhanbetova@shokan.edu.kz; 7 O.A. BaikonurovMining and Metallurgical Institute, Departament of Materials Science and Engineering Physics, Satbayev University, Almaty 050013, Kazakhstan; a.s.beisebayeva@satbayev.university; 8 Departament of Mathematical and Computer Modelling, International Information Technology University, 34/1 Manas Street, Almaty 050040, Kazakhstan; g.mamatova@iitu.edu.kz

**Keywords:** Co_3_O_4_ nanoparticles, solution combustion synthesis, energy storage, supercapacitor, batteries

## Abstract

Co_3_O_4_ nanoparticles synthesized by solution combustion synthesis present a versatile platform for the development of porous nanostructures with tunable morphology and physicochemical properties. Synthesis conditions and parameters such as fuel type; fuel-to-oxidizer ratio and temperature control lead yielding; and Co_3_O_4_ NPs with fine particle size, surface area, and porosity result in enhancing their electrochemical and catalytic capabilities. This review evaluates present studies about SCS Co_3_O_4_ NPs to study how synthesis parameter modifications affect both surface morphology and material structure characteristics including porosity features, which make their improved performance ideal for lithium-ion batteries and supercapacitors. Moreover, the integration of dopants with carbon-based hybrid composites enhances material conductivity and stability by addressing both capacity fading and low electronic conductivity concerns. This review mainly aims to explore the significant relation between fundamental material design principles together with practical uses and provides predictions about future research advancements that aim to enhance the performance of Co_3_O_4_ NPs in next-generation energy and environmental technology applications.

## 1. Introduction

Cobalt (II, III) oxide nanoparticles (Co_3_O_4_ NPs) have emerged as a significant focus in materials science due to their unique properties and versatility. The growing research interest in Co_3_O_4_ NPs is largely attributed to their potential in various applications, including sensitive gas detection sensors [1,2], catalysis [3], and energy storage systems [4,5]. These nanoparticles exhibit a high density of 6.11 g/cm^3^, magnetic behavior, p-type semiconducting characteristics, and notable thermal stability [6,7]. Co_3_O_4_ nanoparticles carry positive charge carriers and exhibit a melting point of 895 °C. Various synthesis methods—such as sol–gel [8], hydrothermal synthesis [9], thermal decomposition [10], co-precipitation [11,12], solution combustion synthesis (SCS) [13], green synthesis [14,15], and electrospinning [16]—have been employed to produce Co_3_O_4_ NPs and tailor their physicochemical properties.

**Figure 1:**
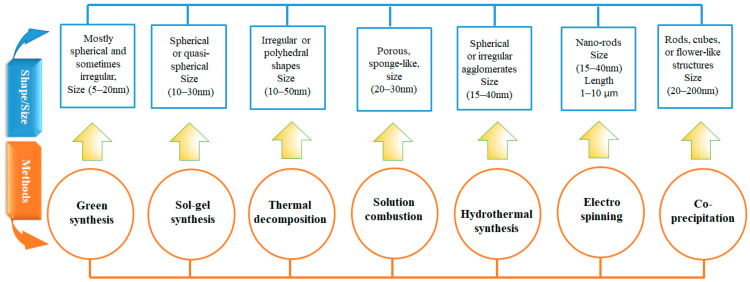
Comparison of typical morphologies and size ranges of Co_3_O_4_ nanoparticles synthesized using various methods, highlighting the influence of synthesis route on particle shape, size, and structural form.

Among these, the SCS method stands out due to its rapid reaction rate, energy efficiency, and ability to produce Co_3_O_4_ nanostructures with high porosity and a more uniform particle size distribution [17,18]. A graphical comparison is presented in Figure 1, illustrating the dimensions and morphologies of Co_3_O_4_ NPs synthesized via different techniques.

Recent studies show that sol–gel and co-precipitation methods typically yield spherical particles ranging from 10 to 40 nm [8], while hydrothermal synthesis produces rod-like or flower-like structures ranging from 2 to 200 nm. In contrast, SCS generally results in porous, agglomerated particles within the 10 to 30 nm range [13,18].

The morphology of nanoparticles plays a critical role in their performance, as surface area directly influences catalytic activity, sensor sensitivity, and energy storage capacity.

**Figure 2:**
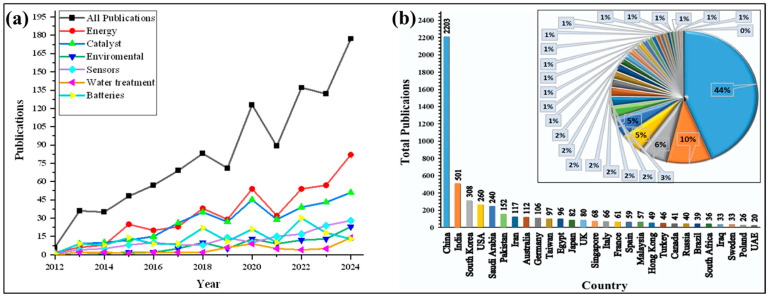
(**a**) Recent publications of Co_3_O_4_ NPs per year from 2012 to 2024 in various potential applications and (**b**) recent publications of Co_3_O_4_ NPs from different countries (data retrieved from the ‘Scopus’ database on 12 April 2025 using the terms ‘Co_3_O_4_ NPs, Batteries, Catalyst, Energy, Environmental, Water treatment and applications’ to search within article title, abstract, and keywords).

Furthermore, the broad applicability of Co_3_O_4_ nanoparticles in fields such as catalysis, sensing, and energy underscores their rapidly growing importance. An overview of the increasing number of publications related to Co_3_O_4_ NPs from 2012 to the end of 2024 is presented in Figure 2a, illustrating the rising scientific interest in these materials over the past decade.

Researchers have increasingly focused on synthesizing Co_3_O_4_ NPs via the solution combustion synthesis (SCS) method, investigating various reaction conditions and parameters to harness its significant advantages. The functional and morphological characteristics of Co_3_O_4_ NPs are closely linked to the synthesis parameters of the SCS method, which are primarily determined by the fuel-to-oxidizer ratio.

Scientific studies have confirmed that altering the fuel-to-oxidizer (F/O) ratio directly influences nanoparticle porosity, particle size, and surface area [19,20]. To tailor Co_3_O_4_ NPs for specific applications, further investigation into their crystalline structure and the factors affecting it is essential.

The SCS method continues to garner significant interest due to its simplicity, cost-effectiveness, and ability to manipulate material properties. This technique operates as a self-propagating high-temperature synthesis process, enabling the rapid formation of metal oxide nanomaterials through exothermic redox reactions between metal precursors and organic fuels in aqueous solutions [21].

Originally, the SCS method was employed by Indian scientist Patil for the synthesis of α-alumina, and it later inspired extensive research into nanostructured metal oxides, including Co_3_O_4_ NPs [22].

The increasing number of studies utilizing the SCS method for Co_3_O_4_ nanoparticle synthesis spans multiple countries and regions, as shown in Figure 2b. This growing body of research highlights the critical influence of synthesis parameters—such as precursor selection, reaction temperature, and the F/O ratio—on key material properties, including morphology, crystallinity, and porosity [18].

Recent studies on critical synthesis conditions and parameters have enabled the tailored production of Co_3_O_4_ nanoparticles with promising properties for use as anodes in lithium-ion batteries [23]. Their potential as electrocatalysts for oxygen evolution reactions (OER) in alkaline media further supports their role in future sustainable energy conversion technologies [24].

The performance of Co_3_O_4_ NPs in these systems is largely attributed to their porous structure, which provides a high active surface area and facilitates efficient ion transport. Their redox activity and stability also make them suitable as electrode materials for supercapacitor applications in next-generation energy storage systems [25].

Previous research has compared the SCS method with metal–organic framework (MOF) decomposition, revealing differences in particle structure and synthesis efficiency [26]. Additionally, electrochemical sensors based on Co_3_O_4_ NPs exhibit high sensitivity and selectivity, enabling the detection of various chemicals—including hydroquinone—demonstrating their value in analytical applications.

Current applications of Co_3_O_4_ nanoparticles extend beyond energy and sensing domains into environmental remediation. Functionalized forms, such as biochar-impregnated Co_3_O_4_ NPs, have been effectively used for the removal of pharmaceutical contaminants from water sources [27]. Plant-based biosynthesis methods are gaining popularity due to their environmentally friendly nature and their potential for integrating Co_3_O_4_ NPs into agricultural systems [28].

Studies investigating the interaction between Co_3_O_4_ NPs and plants have reported mixed results, particularly regarding their impact on plant physiology through altered antioxidant activity and ion transport mechanisms [29]. Additionally, Co_3_O_4_ NPs synthesized via thermal decomposition using solar energy have demonstrated superior photocatalytic performance, confirming their potential for environmental cleanup applications [10].

Advancements and modifications in synthesis techniques have demonstrated that Co_3_O_4_ nanoparticles can be effectively utilized in a range of advanced applications. For example, a hybrid precipitation–hydrothermal synthesis method combined with the surfactant cetyl-trimethylammonium bromide (CTAB) was employed to enhance the photocatalytic performance of Co_3_O_4_ NPs, providing strong evidence of their potential for environmental remediation [30]. Moreover, incorporating Co_3_O_4_ nanorods into carbon nanofibers via electrospinning enhances their one-dimensional morphology, increases surface area, and improves conductivity, thereby facilitating their application in advanced supercapacitors [16]. Similarly, Yan et al. (2025) investigated hierarchical Co_3_O_4_/ZnIn_2_S_4_ heterostructures, demonstrating their high efficiency for hydrogen generation under visible light illumination [31]. The development of a Co_3_O_4_/reduced graphene-oxide-based sensor for sensitive Pb (II) detection represents a significant advancement in heavy metal detection research.

Furthermore, Co_3_O_4_ nanoparticles modified with multi-walled carbon nanotubes (MWCNTs) in polyvinylidene fluoride (PVDF) composites exhibited notable thermal and electrical enhancements, highlighting their potential for flexible electronic devices [32]. The biomedical potential of Co_3_O_4_ NPs as antimicrobial and anticancer agents was confirmed by Ifijen et al. (2025) [33].

Additionally, researchers have improved the gas-sensing capabilities of Co_3_O_4_ NPs by developing two novel methods: electrospray deposition for acetone detection [34] and microwave-assisted synthesis for methane detection [35].

The main aim of this review is to provide a comprehensive overview of the synthesis strategies, morphological evolution, and multifunctional applications of Co_3_O_4_ nanoparticles, with a particular focus on the synthesis conditions and parameters of the solution combustion synthesis (SCS) method. By critically analyzing previous advancements and investigations, this review elucidates the properties, synthesis processes, characterization techniques, and applications of Co_3_O_4_ NPs, alongside a comprehensive comparison of prior studies. This approach fosters a deeper understanding of recent developments and potential future directions.

Additionally, this review offers a roadmap for future research by highlighting current challenges and innovative strategies, thereby assisting emerging researchers in addressing these challenges and bridging existing knowledge gaps. Ultimately, this will enhance and mobilize the transformative potential of Co_3_O_4_ nanoparticles across multidisciplinary technologies.

## 2. Materials and Methods

This review provides a critical overview of the production of Co_3_O_4_ NPs via the SCS method, highlighting the impact of various synthesis parameters on their structural and functional attributes, including shape and size characteristics. A comprehensive literature search was conducted using peer-reviewed databases including Web of Science, Scopus, ScienceDirect, and Springer Nature Link. The search employed specific keywords such as “Co_3_O_4_ nanoparticles”, “solution combustion synthesis”, “fuel-to-oxidizer ratio”, “porous Co_3_O_4_”, and “nanostructures for energy applications”. Selection criteria focused on research articles published between 2010 and 2025 that detailed the synthesis of Co_3_O_4_ NPs via the SCS method, providing thorough information on synthesis conditions, structural characterization, and application performance.

Studies employing alternative synthesis methods or lacking experimental results were excluded. The selected articles were critically analyzed and categorized based on synthesis parameters, nanoparticle morphology, and targeted applications, thereby providing both qualitative depth and quantitative insight into the field.

The SCS synthesis method is widely used for the synthesis of Co_3_O_4_ NPs due to its scalability and simplicity. In this method, the initial precursors typically consist of powdered volatile oxidizers—such as cobalt salts like nitrates and chlorides—and fuels including glycine, cellulose, citric acid, urea, or plant-based extracts, all of which are highly soluble in water [36]. SCS method involves decomposition of the precursors, fuel oxidation, and combustion reaction. The SCS process requires an exothermic reduction reaction of a prepared precursor solution, which leads to both dehydration of precursor components for the synthesis of Co_3_O_4_ NPs within a brief period.

Cobalt nitrate hexahydrate as an oxidant includes the following steps: firstly, cobalt nitrate hexahydrate decomposes upon heating to produce cobalt oxide species according to Equation (1). Citric acid acts as a reducing agent and is oxidized during the combustion process (2). During fuel combustion, oxygen released from cobalt nitrate reacts with carbon monoxide (CO) produced by the fuel, sustaining the burning reaction (3). Finally, the high-temperature conditions facilitate the synthesis of Co_3_O_4_ NPs (4) [25]. To synthesize cobalt oxide, cobalt nitrate hexahydrate (Co(NO_3_)_2_·6H_2_O) must be prepared in precise amounts, typically with fuel-to-oxidizer ratios (φ) of 0.5, 1.0, 1.5, or 2.0, depending on the desired properties of the resulting Co_3_O_4_ nanoparticles [19,23].

The oxidizer (cobalt nitrate hexahydrate) needs to be dissolved in deionized water to form a clear solution, and the calculated amount of the fuel should be added to the prepared oxidizer solution. The solution was thoroughly stirred to ensure complete dissolution of the oxidizer and fuel. The precursor solution was then placed on a hotplate to initiate the combustion reaction. This prepared solution is heated in air at temperatures ranging from approximately 300 to 900 °C, triggering an exothermic reaction that results in the synthesis of Co_3_O_4_ NPs [18,19,23]. Filtration processes suggested for purification of obtained Co_3_O_4_ NPs to remove residual reactants and impurities. Obtained particles can be washed with ethanol or deionized water and dried at a moderate 70 to 80 °C temperature for 24 h. Optionally, annealing of the dried NPs is recommended for enhancing the crystallinity and structural stability [19].

(1) $$2\mathrm{Co}{\left(NO_{3}\right)}_{2}\cdot 6H_{2}O\overset{\mathrm{Heat}}{\to }2CoO+4NO_{2}+12H_{2}O+O_{2}$$

(2) $$2C_{6}H_{8}O_{7}+6O_{2}\overset{\mathrm{Combustion}}{\to }6CO_{2}+8H_{2}O+6CO$$

(3) $$6CO+6NO_{2}\overset{\mathrm{High}\;\mathrm{Temperature}}{\to }6CO_{2}+3N_{2}+3O_{2}$$

(4) $$6\mathrm{CoO}+4O_{2}\overset{\mathrm{Heat}}{\to }2{\mathrm{Co}}_{3}O_{4}$$

**Figure 3:**
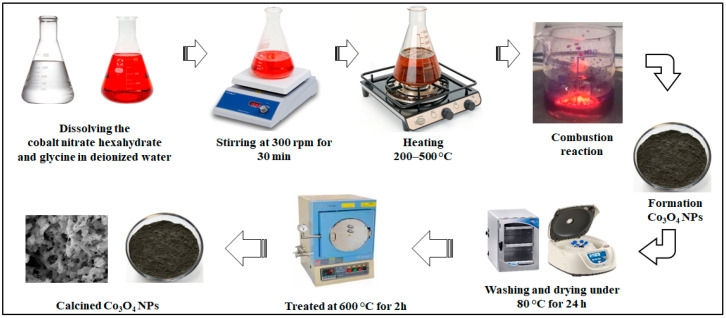
Schematic representation of the process steps involved in the synthesis of Co_3_O_4_ NPs via the solution combustion synthesis SCS method.

The rapid generation of gases such as CO_2_, H_2_O, and N_2_ during the combustion process helps create porous structures with a high surface area, which are advantageous for electrochemical and catalytic applications. A schematic diagram in Figure 3 illustrates the typical stages of the solution combustion synthesis process, beginning with precursor preparation, followed by dehydration, auto-ignition, and combustion, and concluding with post-calcination treatments.

Numerous scientific studies have demonstrated the effective use of this method to produce nanostructured Co_3_O_4_ materials with well-controlled properties. For example, researchers have synthesized nanoscale Co_3_O_4_ using glycine as both a fuel and a complexing agent, yielding materials with crystallite sizes below 25 nm, which are well-suited for use as lithium-ion battery anodes [23].

Experiments using different fuel mixtures and F/O ratios with urea demonstrated that fuel-rich solutions produced smaller, more porous particles, while fuel-lean conditions resulted in denser and larger morphologies [18]. Laboratory studies found that the optimal F/O ratio ranged between 0.5 and 2.0, balancing combustion efficiency with crystallinity and porosity [19]. Additionally, controlled combustion conditions and precursor concentrations led to the formation of hollow Co_3_O_4_ nanospheres, which exhibited enhanced specific capacitance, making them promising for supercapacitor applications [37].

A summary of synthesis parameters for comparative analysis is presented in Table 1 whereby the reviewed literature presents information about fuel type, F/O ratio, synthesis temperature, and particle size and morphology. The collected data demonstrates that particular changes in combustion parameters substantially shape both Co_3_O_4_ NPs structure along with their respective application properties, which reveals vital process, structure, and function associations for optimized Co_3_O_4_ NPs across energy storage and catalytic and sensing applications.

## 3. Cobalt (II) (III) Oxide (Co_3_O_4_) Nanoparticles

Co_3_O_4_ nanoparticles have emerged as highly functional nanomaterials due to their versatile chemical nature; unique spinel crystal structure; and broad range of applications in catalysis, energy storage, sensing, and biomedical fields. Co_3_O_4_ crystallizes in a normal spinel structure where Co^2+^ ions occupy tetrahedral sites and Co^3+^ ions occupy octahedral sites within the cubic lattice. This mixed valence state imparts redox flexibility [39]. In contrast, CoO has a rock salt structure with Co^2+^ ions located only in octahedral sites, resulting in limited redox flexibility and reduced catalytic activity. CoO also exhibits structural instability and poor cycling performance in energy storage applications [40]. CoO_2_, often appearing as Co_2_O_4_ in nonstoichiometric systems, adopts a layered hexagonal or rutile-type structure derived from Co^4+^ ions, which provides high oxidizing power but suffers from poor stability and structural degradation during redox cycling [41].

Therefore, Co_3_O_4_ is preferred for various applications due to its stable structure, mixed valence states, and superior electron conductivity. The stoichiometry of Co_3_O_4_—three cobalt atoms and four oxygen atoms per formula unit—contributes to excellent electronic conductivity and catalytic activity [42]. Various synthesis methods produce Co_3_O_4_ nanoparticles in the 5–50 nm size range, significantly influencing their surface area, chemical reactivity, and electronic properties.

The catalytic and electrochemical performance of Co_3_O_4_ nanoparticles is significantly enhanced by their high surface-to-volume ratio, which exposes more active reaction sites. Studies have reported that Co_3_O_4_ nanoparticles can achieve excellent oxygen evolution reaction (OER) performance, with overpotentials as low as 290 mV at a current density of 10 mA cm^−2^, highlighting their potential in electrocatalysis [43]. Additionally, their high theoretical capacitance of 3560 F g^−1^, resulting from reversible redox transitions between Co^2+^, Co^3+^, and Co^4+^ oxidation states, makes Co_3_O_4_ nanoparticles highly promising for energy storage applications [44].

The chemical properties of Co_3_O_4_ nanoparticles include a moderate band gap ranging from 1.6 eV to 2.2 eV, enabling their use in photocatalysis and optoelectronic devices. Their reactivity is strongly influenced by crystallinity, particle size, and surface defect density, which can be tuned through various synthesis methods. Recent studies have shown that phytosynthesized Co_3_O_4_ nanoparticles exhibit potent antibacterial and antioxidant properties, offering promising prospects for biomedical applications such as drug delivery systems and wound healing [45]. Furthermore, Co_3_O_4_ nanoparticles demonstrate thermal stability up to 900 °C and exhibit magnetic ordering at 30 K, making them suitable candidates for magnetic storage devices [39].

The surface of Co_3_O_4_ nanoparticles can be modified or doped to enhance selectivity and reactivity in catalytic applications. They also hold significant potential for driving innovation in future technologies. Hybrid composites containing Co_3_O_4_ nanoparticles, as well as heterostructures and multi-metal oxide systems incorporating these nanoparticles, are expected to advance developments in hydrogen generation, carbon dioxide reduction, and advanced battery technologies [43]. By carefully controlling synthesis conditions and parameters such as temperature, fuel-to-oxidizer ratio, and precursor selection, Co_3_O_4_ nanoparticles can be tailored to address current energy and environmental challenges effectively [45].

### Characteristics

**Figure 4:**
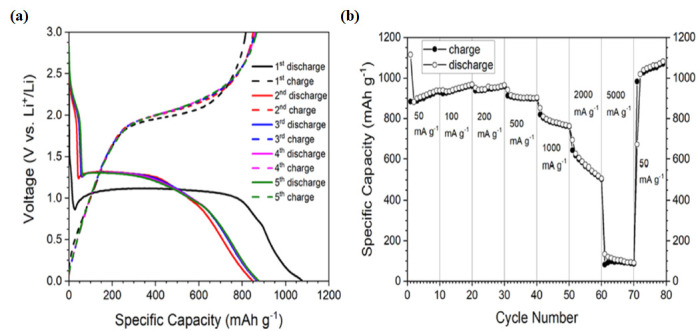
Electrochemical characteristics for Co_3_O_4_ NPs electrodes: (**a**) charge–discharge profiles from the first to fifth cycles tested at a current density of 1000 mA·g^−1^; (**b**) rate capability tests at various current densities ranging from 50 to 5000 mA·g^−1^ (Figure (**a**,**b**) reproduced from [23] © 2021 published by Beilstein-Institutli).

Co_3_O_4_ nanoparticles exhibit excellent electrochemical performance and are widely used as electrode materials in lithium-ion batteries. As shown in Figure 4a,b, Co_3_O_4_ nanoparticles demonstrate a specific capacity of 1060 mAh g^−1^, achieved after 100 cycles at a current density of 1000 mA g^−1^. Additionally, they show impressive cycling stability across a range of current densities between 50 and 5000 mA g^−1^ [23].

The electrochemical performance of Co_3_O_4_ powder as an anode material was evaluated using coin-type electrodes within a voltage window of 0.01 to 3.0 V vs. Li^+^/Li. Different coin cells exhibited very similar behavior, attributed to the stabilized reversible conversion and reconversion reactions [46]. Figure 4a presents the charge–discharge profiles from the first to fifth cycles at a current density of 100 mA g^−1^. The initial discharge and charge capacities are approximately 1075 and 817 mAh g^−1^, respectively.

The initial Coulombic efficiency is around 76%, which reflects a typical capacity loss of about 25% during the first cycle in Co_3_O_4_ nanoparticle electrodes. This phenomenon has been well documented for Co_3_O_4_ nanostructures with various morphologies [43,44]. The charge profiles exhibit a voltage plateau near 2.0 V, primarily corresponding to the reconversion reaction and the subsequent structural reconstruction of the cobalt oxide electrode.

The shapes of the second and subsequent charge–discharge curves are nearly identical, indicating a high cycling stability of the Co_3_O_4_ NP electrode during lithiation/delithiation processes [21]. Given that high-rate capability is a critical requirement for high-power applications, rate performance measurements were conducted at current densities ranging from 50 to 5000 mA·g^−1^. The results, shown in Figure 4b, demonstrate a significant decline in both charge and discharge capacities at current densities above 500 mA·g^−1^.

It is evident that the capacities experience a slight initial fading over the first few cycles, followed by stabilization at specific levels—namely, 898 mAh·g^−1^ at 500 mA·g^−1^, 761 mAh·g^−1^ at 1 A·g^−1^, 505 mAh·g^−1^ at 2000 mA·g^−1^, and 90 mAh·g^−1^ at 5000 mA·g^−1^, respectively.

(5) $${\mathrm{Co}}_{3}O_{4}+8Li^{+}+8e^{-}\overset{\mathrm{discharge}}{\underset{\mathrm{charge}}{⇄}}3Co+4Li_{2}O$$

When the current rate is rapidly increased to 50 mA·g^−1^ after cycling at higher current densities, the charge and discharge capacities continue to rise and stabilize at approximately 1070 mAh·g^−1^. This indicates that the Co_3_O_4_ nanoparticle anode studied here exhibits excellent rate capability and reversibility. This performance is attributed to the unique arrangement of the particles, which shortens the diffusion paths for Li^+^ ions and conversion reactions while providing efficient, electrolyte-accessible pathways for ion transport [23]. As a result, Co_3_O_4_ nanostructures synthesized by the SCS method demonstrate superior electrochemical performance compared to nanoparticles reported in earlier studies.

The electrochemical analysis reported by Afrooze et al. (2024) [47] showed that Co_3_O_4_ NPs exhibited a capacitance of 603 F g^−1^ at a scan rate of 1 mV s^−1^ and maintained excellent stability, retaining more than 97.6% of their capacity even after 5000 charge–discharge cycles. These findings suggest their strong potential for developing high-performance and durable lithium-ion batteries [48]. The underlying electrochemical mechanism is based on a conversion reaction (Equation (5)), which allows for the reversible storage of up to eight lithium ions. Multiple studies have also highlighted Co_3_O_4_ nanoparticles as effective electrocatalysts due to their abundance; low cost; environmental sensitivity; and, in some cases, performance comparable to that of noble-metal-based materials [49]. Furthermore, Co_3_O_4_ NPs produced with an optimized amount of oxidizer and fuel (i.e., when the ratio of cobalt nitrate and ascorbic acid is 1:1) have displayed better catalytic performance for oxygen evolution reaction. Maurya et al. (2023) synthesized Co_3_O_4_ nanoparticles by introducing an interlayer of copper to modify their reactivity toward the oxygen evolution reaction (OER) [50]. This synthesis strategy aimed to enhance the electrochemical performance of Co_3_O_4_ nanoparticles for improved OER activity.

The number of precursors in the samples was found to influence the reduced activity associated with the copper interlayer. This effect is attributed to the presence of Co^3+^ ions on the surface, which impacts the catalytic behavior. In a related study, Vennela et al. (2019) analyzed the crystallite size and structure of Co_3_O_4_ nanoparticles and confirmed that their lattice parameters align with those expected for a spinel structure, thereby confirming the crystalline stability of the material [51]. Cyclic voltammetry (CV) and galvanostatic charge–discharge (GCD) methods were employed to evaluate the electrochemical characteristics of Co_3_O_4_ nanoparticles, including capacitance and charge–discharge behavior [25,48,52]. For example, CV was conducted within a potential window of −0.30 to 0.45 V vs. SCE, and GCD was performed within a potential window of 0 to 0.35 V vs. SCE, using a CHI660D electrochemical workstation (Shanghai, China).

The specific capacitance can also be calculated from the CV data using Equation (6). Furthermore, electrochemical impedance spectroscopy (EIS) was conducted to analyze the charge transport properties. EIS measurements were carried out over a frequency range of 100 kHz to 10 MHz at open-circuit voltage, using an AC perturbation of 10 mV on a Cs 350 electrochemical workstation (Wuhan, China) [52,53].

(6) $$C=\frac{1}{mv(Va-Cc)}\int_{Va}^{Vc}\;I\left(V\right)dV$$

At the same time, the specific capacitance can also be calculated from GCD data using Equation (7) [9,54].

(7) $$C=\frac{i\times △t}{△V\times m}$$

**Figure 5:**
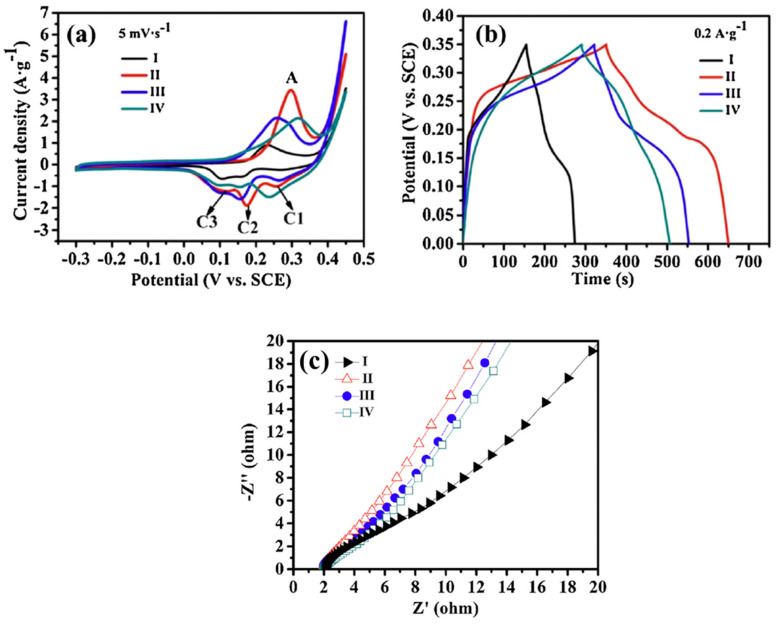
(**a**) CV curves of samples (I, II, III, and IV) at the scan rate of 5 mVs^−1^. (**b**) Galvanostatic charge–discharge curves of samples I, II, III, and IV at a constant discharge current density of 0.2 Ag^−1^. (**c**) Nyquist plots of experimental impedance data for sample II, II-350, II-450, and II-550 electrodes. This figure is reprinted from [25], with permission from Elsevier B.V., 2014.

Specific capacitance is one of the most critical parameters for evaluating electrochemical performance. Based on Equation (6), the specific capacitance values of samples I, II, III, and IV were calculated from the CV curves (Figure 5a) to be 68.3, 155.3, 146.8, and 135.7 F·g^−1^, respectively. Correspondingly, the GCD data (Figure 5b) showed specific capacitance values of 73.1, 179.7, 141.6, and 130.5 F·g^−1^ at a current density of 0.2 A·g^−1^ for samples I, II, III, and IV, respectively. These values are consistent with those obtained from the CV analysis.

Both CV and GCD measurements indicated that sample II exhibited the best capacitive performance among the tested samples. The Nyquist plots of experimental impedance data for samples I–IV are shown in Figure 5c. The internal resistance of the electrodes—comprising the contact resistance at the active material/current collector interface, the intrinsic resistance of the active material, and the ionic resistance of the electrolyte—was approximately constant across all samples (2.0 Ω), as indicated by the intercept on the x-axis [55].

In the linear region of the Nyquist plots, the slope was approximately 60° for samples II–IV and 45° for sample I. A 45° line typically reflects higher resistance due to limited electrolyte diffusion within the electrode, while a 60° slope is more indicative of capacitive behavior (with an ideal capacitor exhibiting a vertical line) [56]. This suggests that samples II–IV demonstrate higher supercapacitive performance and better hydroxyl ion accessibility at the electrode surface. From a microstructural perspective, mesoporous structures and high specific surface area (SSA) provide shorter ion-transport pathways, which help reduce Warburg resistance [57,58,59]. Sample II showed the highest SSA and mesopore volume, as confirmed by BET analysis, thereby offering the lowest Warburg resistance and best supercapacitive performance [25].

**Figure 6:**
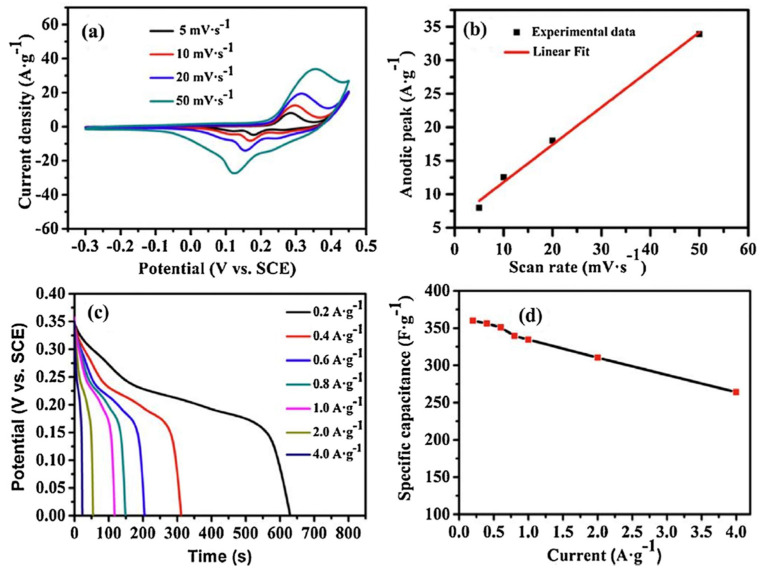
(**a**) Cyclic voltammetry (CV) profiles of sample II-350 recorded at different scan rates; (**b**) plot showing the linear relationship between anodic current density and scan rate, indicating a surface-controlled pseudocapacitive process; (**c**) galvanostatic charge–discharge (GCD) curves of sample II-350 under various current densities within a potential window of 0–0.35 V; (**d**) variation of specific capacitance as a function of current density, derived from the GCD data, demonstrating excellent rate capability. This figure is reprinted from [25], with permission from Elsevier B.V., 2014.

The CV curves of sample II-350, recorded at scan rates ranging from 5 to 50 mV·s^−1^ in a 6.0 mol·L^−1^ KOH aqueous electrolyte over a potential window of 0.3 to 0.35 V, are shown in Figure 6a. The non-rectangular shape of the CV curves confirms a pseudocapacitive charge storage mechanism involving reversible redox reactions. With increasing scan rate, the anodic and cathodic peaks shifted to higher and lower potentials, respectively, which is attributed to electrode polarization effects [60,61]. Anodic peak current density and scan rate exhibited an almost linear (quasi-linear) relationship (Figure 6b), which suggested surface redox processes related to the electrode’s pseudo-capacitance behavior. High-rate discharge capability and exceptional cycling stability are crucial characteristics for electrode materials used in supercapacitors [62].

The rate performance of Co_3_O_4_ nanoparticles was investigated using GCD curves recorded at various current densities over a potential range of 0–0.35 V, with the results presented in Figure 6c. The pseudocapacitive behavior of Co_3_O_4_ NPs is illustrated in Figure 6d, as evidenced by the nonlinearity of the discharge curves—consistent with the behavior observed in the CV tests. The specific capacitance of the sample II-350 Co_3_O_4_ electrode ranged from 362.8 to 285.7 F·g^−1^ as the current density increased from 0.2 to 4 A·g^−1^. Furthermore, the sample demonstrated excellent rate performance, retaining approximately 78.7% of its initial capacitance even at the high current density of 4 A·g^−1^.

## 4. Results and Discussion

Altering synthesis conditions in the solution combustion synthesis (SCS) process of Co_3_O_4_ nanoparticles is crucial for tailoring their properties, including particle size, morphology, and overall performance. As summarized in Table 2, numerous researchers have investigated these parameters to optimize the nanoparticles for specific applications. Control over the average particle size, typically ranging from 12 to 64 nm, has been effectively achieved by adjusting the ignition temperature between 300 °C and 800 °C [63]. In addition to Co_3_O_4_, other spinel oxides such as CoFe_2_O_4_, NiFe_2_O_4_, and Co_0.5_Ni_0.5_Fe_2_O_4_ have also been synthesized via the SCS method. These materials exhibit pure cubic spinel structures, and by tuning the fuel-to-oxidizer (F/O) ratio, researchers have enhanced their magnetic properties, making them highly suitable for biomedical applications [64,65].

Magnetic measurements revealed that Co_3_O_4_ nanoparticles primarily exhibit antiferromagnetic behavior, with the Néel temperature decreasing as the average particle size shrinks from 200 to 400 nm down to 5–18 nm [63,64]. Conversely, lower synthesis temperatures also influence particle size; at temperatures around 190–240 °C, very small Co_3_O_4_ nanoparticles, ranging from 2.3 to 7.4 nm, can be obtained due to the high loading of Co_3_O_4_ (up to 59%) within a carbon network [66]. In contrast, increasing the synthesis temperature generally results in larger nanoparticle sizes, enhanced porosity, and improved electronic conductivity.

**Table 2:** A comparison of synthesized Co_3_O_4_ NPs through the SCS method in different conditions with their properties.

No.	Used Precursors and Fuel Solution	Electrolyte	Specific Capacitance, Fg^−1^	Surface Area/m^2^g^−1^	Pure Volume/cm^3^g^−1^	T_A_, °C	Reaction T, °C	Particle Size/Diameter, nm	Proposed Applications	References
1	(Co(NO_3_)_2_·6H_2_O) and (CO(NH_2_)_2_) as fuel	Alkaline	1060	3	0.02	600		36	As the anode material for Li-ion batteries	[23]
2	(Co(NO_3_)_2_·6H_2_O) and glycine, NH_2_CH_2_COOH	1 M KOH		10.45		300	500	13.1	Best-performing electrode obtaining	[24]
3	(Co(CH_3_-CO_2_)_2_ 4H_2_O) and urea (CH_4_N_2_O) as fuel					500		70	Catalysis and energy storage applications	[19]
4	Cobalt nitrate hexahydrate and 2-imidazolidinone hemihydrate (ethylenurea)					500		26.0	Sensitive sensors for the safety of environmental and healthcare	[1]
5	(Co(NO_3_)_2_·6H_2_O) and methanol as feul	1 M KOH	3560			500			Electrode for electrochemical applications	[26]
6	5 g (Co(CH_3_- CO_2_)_2_·6H_2_O) and 1.72 g urea (CH_4_N_2_O) as fuel and 15 mL deionized water	KOH				400	900	50	Active for oxygen evolution reaction (OER)	[67]
7	(Co(CH_3_-CO_2_)_2_∙6H_2_O) and citric acid monohydrate (C_6_H_8_O_7_·H_2_O) and ammonium nitrate (NH_4_NO_3_) were used as fuel		362.8	17.9	0.095	350	550	26.1	Supercapaci tors electrode materials	[25]
8	3M(Co(NO_3_)_2_∙6H_2_O), 6M glycine (C_2_H_5_NO_2_), 10% by weight of cobalt nitrate (nitric acid) and 50 mL deionized water		700	90	292.66	260	260	20–65	Gas sensors	[18]
9	Co(NO_3_)_2_⋅6H_2_O and urea, NH_2_CONH_2_ with 100 mL deionized water	3 M KOH	212	69.34	0.0431	600		13.64	High-performance electrodes for supercapaci tors	[47]
10	(CoCl_2_∙6H_2_O), D-glucose, fructose, maltose, sucrose	1 M KOH				600			Non-enzyme glucose detection	[68]
11	Cobalt nitrate, urea as fuel and deionized water			1.4	0.016	400		30–50	In catalysts as coatings	[69]
12	(CoCl_2_∙6H_2_O), (AgNO_3_) and (NH_3_), in deionized water	0.1 M KOH	992.7	407.33	0.1155			12.98	Supercapaci tors application	[11]
13	(CoCl_2_∙6H_2_O), (AgNO_3_) and (NH_3_), in deionized water	0.1 M KOH		53.06	0.07425			19.37	Supercapaci tors application	[11]

Decreasing the fuel-to-oxidizer (F/O) ratio influences both the crystal size and lattice parameters of nanocrystalline Co_3_O_4_ [23,49]. The final properties of Co_3_O_4_ nanoparticles, including grain size and surface area, depend significantly on the F/O molar ratio as well as the calcination temperature [19,49,68]. Moreover, while the SCS method commonly uses cobalt nitrate hexahydrate and citric acid, the combustion reaction can also proceed in an alkaline medium [24,69], leading to the formation of porous Co_3_O_4_ nanoparticles. These porous structures have been reported to exhibit enhanced performance as OER electrocatalysts.

The specific capacitance of Co_3_O_4_ varies significantly depending on the fuel used in the synthesis due to differences in combustion kinetics and resultant porosity. Ethanol (Mw = 46.07 g·mol^−1^) achieved the highest specific capacitance of 3560 F·g^−1^, attributed to its rapid combustion at 500 °C, which produces highly porous and conductive Co_3_O_4_. In contrast, urea and glycine (Mw = 60.06 and 75.07 g·mol^−1^, respectively) yielded lower capacitances of 1060 F·g^−1^ and 700 F·g^−1^ when calcined at 600 °C and 260 °C, respectively. Citric acid (Mw = 192.12 g·mol^−1^), despite its strong thermal stability and complexation capability, resulted in the lowest capacitance value of 362.8 F·g^−1^, likely due to slower gas evolution leading to less effective porosity development.

**Figure 7:**
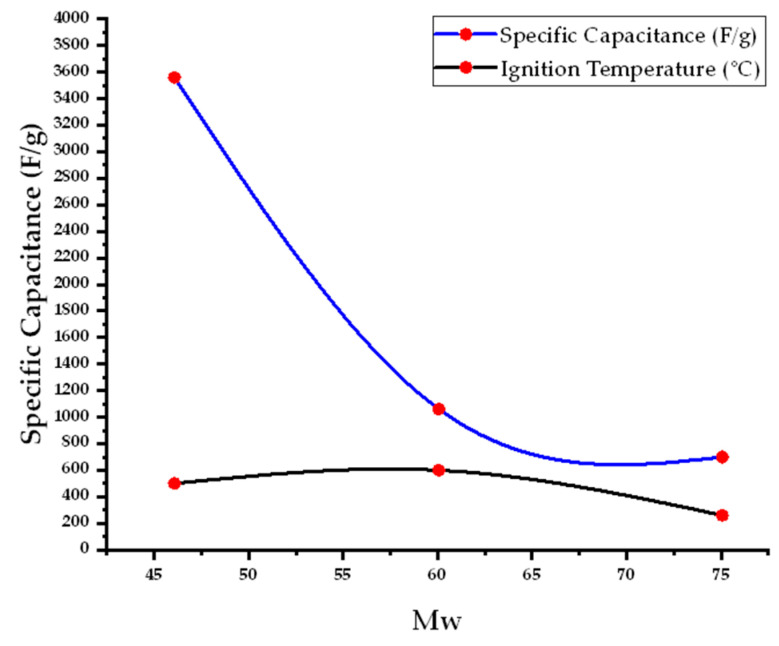
The relationship of fuel molecular mass with the ignition temperature in the process affects the specific capacitance of the Co_3_O_4_ NPs.

The specific capacitance of Co_3_O_4_ NPs synthesized by SCS depends on both the synthesis temperature and the choice of fuel (molecular weight (Mw)). The data in (Figure 7) reveal that fuels with lower molecular weights tend to produce lower ignition temperatures, leading to highly exothermic and rapid reactions that enhance the porosity and surface area of the resulting materials. These structural features significantly improve ion transport and charge storage capability, resulting in higher specific capacitance. In contrast, fuels with higher molecular weights require elevated ignition temperatures, which may promote grain growth and reduce the active surface area, thereby lowering the electrochemical performance. The plot demonstrates the importance of optimizing combustion conditions to maximize supercapacitive performance.

### 4.1. Characterization

**Figure 8:**
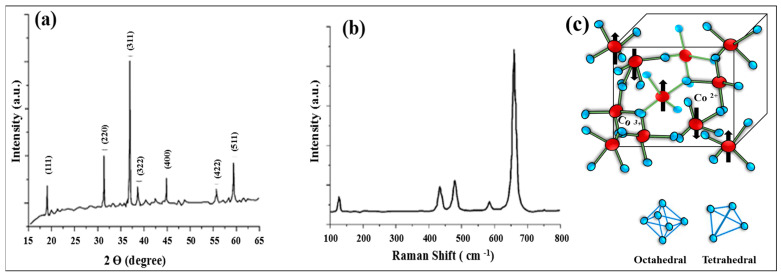
(**a**) XRD pattern; diffractogram of Co_3_O_4_ NPs obtained by the SCS method from a cobalt nitrate–glycine mixture at φ = 1.5. (**b**) Raman spectrum for Co_3_O_4_ powder. (**c**) Schematic representation of the cubic spinel structure of Co_3_O_4_ NPs (Figure (**a**,**b**) was reproduced from [23] © 2021 published by Beilstein-Institut).

Powder X-ray diffraction (XRD) and Raman spectroscopy were employed to analyze the structural features of Co_3_O_4_ NPs, as presented in (Figure 8). First, the Co_3_O_4_ NPs were found to have a crystalline nature based on the XRD pattern. In Figure 8a, the X-ray diffraction pattern reveals seven peaks at 19°, 31.3°, 36.8°, 38.7°, 44.8°, 55.8°, and 59.4°, corresponding to the (111), (220), (311), (222), (400), (422), and (511) crystal planes, respectively [23]. These peak positions confirm the cubic spinel crystal structure with the space group Fd3m. The calculated unit cell parameter (a = 8.085 Å) is consistent with the standard value for Co_3_O_4_ NPs. According to the Scherrer formula, analysis of peak broadening yielded an estimated average crystallite size of around 40 nm. In Figure 8b, five distinctive Raman active vibrational modes are clearly identified: F2g3 at 184 cm^−1^, Eg at 464 cm^−1^, F2g2 at 506 cm^−1^, F2g1 at 601 cm^−1^, and A1g at 670 cm^−1^ [70]. The band at 670 cm^−1^ corresponds to the Co–O symmetric stretching vibration of octahedral CoO_6_ units, whereas the band at 184 cm^−1^ is related to tetrahedral CoO_4_ sites. The other peaks correspond to mixed motions of oxygen atoms at tetrahedral and octahedral sites [71]. The tetrahedral sites in Co_3_O_4_ NPs contain Co^2+^ ions, while the octahedral sites are occupied by Co^3+^ ions, making the material both redox-active and mixed-valence, as illustrated in Figure 8c. For more detailed information on the electronic structure and chemistry of Co_3_O_4_ NPs, X-ray photoelectron spectroscopy (XPS) and X-ray absorption spectroscopy (XAS) analyses are required.

Specifically, the deconvolution of the O 1s XPS peak provides information about the oxygen species on the surface, generally showing lattice oxygen (529.5 eV), oxygen vacancies (531.0 eV), and surface oxygen or hydroxyl groups (532.5 eV) [72], which are crucial for both oxidation and electron transport. The combination of Co_3_O_4_ NPs with mesoporous carbon leads to an approximately 11% increase in non-lattice oxygen species on the surface, which, in turn, enhances both wettability and electrochemical performance [66].

In addition to O 1s XPS, Co 2p XPS confirms the presence of both Co^2+^ and Co^3+^ in the structure, demonstrated by peaks at approximately 780 eV and 795 eV for Co 2p [65]. By analyzing both the O-K and Co-L edges in XAS, it was found that there was a hybridization of O 2p and Co 3d orbitals, which helps elucidate the mechanism of charge transfer and the presence of defects in the sample [72].

Scanning electron microscopy (SEM) and transmission electron microscopy (TEM) have been widely used to observe the surface morphology and particle size [47,69]. It was concluded from the SEM and TEM images that the cobalt oxide particles ranged from 12 to 60 nm, with an average size of about 36 nm, and exhibited a loose arrangement with several void spaces [23].

**Figure 9:**
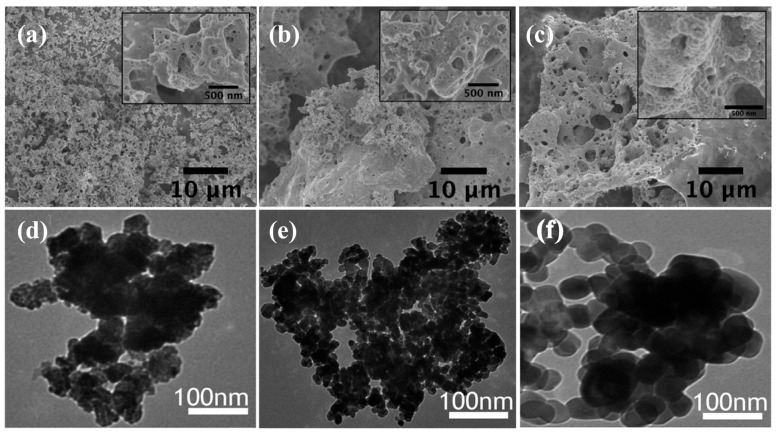
SEM images (**a**–**c**) reveal highly porous Co_3_O_4_ nanostructures with interconnected macro- and mesopores [24], with permission from Elsevier B.V., 2014. TEM images (**d**–**f**) show aggregated, quasi-spherical nanoparticles with average sizes of 8 to 12 nm and clear lattice fringes, confirming the crystalline spinel phase [73], with permission from Elsevier B.V., 2016.

SEM and TEM images in Figure 9 confirm the XRD and Raman analysis results and illustrate significant morphological and structural characteristics of Co_3_O_4_ NPs. SEM imaging (Figure 9a–c) revealed a highly porous structure with interconnected macropores visible in both the main images and their insets. The combustion synthesis process involves rapid gas evolution and a self-sustained reaction that creates pores ranging from 100 nm to 1 µm in size [24,25]. This hierarchical pore structure facilitates faster ion transfer and improved electrolyte accessibility to electrode surfaces during electrochemical processes [73]. The inset magnifications emphasize the nanostructured skeletal framework, confirming the multi-scale porosity essential for increasing the surface-to-volume ratio. Such a structure significantly enhances charge storage capacity in supercapacitors and boosts electrocatalytic activity by providing a greater number of exposed active sites [74,75].

Transmission electron microscopy images (Figure 9d–f) revealed the nanoscale arrangement and crystalline properties of Co_3_O_4_ particles. The microstructure observed in Figure 9d,e shows dense nanosheets interspersed with hollow regions, characteristic of combustion synthesis products obtained under fuel-rich conditions. These porous nanoclusters enhance surface contact with electrolytes and effectively increase the system’s surface area, benefiting energy storage and catalytic applications [76]. The nanoparticles in Figure 9f display a uniform distribution of quasi-spherical structures ranging from 8 to 12 nm in size. The narrow size distribution observed in the synthesized crystals results from controlled nucleation and suppressed growth, achieved through optimized fuel-to-oxidizer ratios during the SCS synthesis.

The small-scale nature of these crystallites enhances electrical conductivity and pseudocapacitive performance in electrochemical applications by enabling faster redox processes and reducing ion transport distances [73,74]. Figure 9f displays HRTEM images showing distinct lattice fringes with spacings of 0.24–0.28 nm, corresponding to the (311) and (220) planes of the Co_3_O_4_ spinel phase. The strong and uniform fringes observed indicate that the material possesses high crystallinity with minimal amorphous content, which supports structural stability and improves electrochemical performance [75,77]. These clear lattice planes confirm that the functional Co_3_O_4_ NPs used in catalysis and energy devices have formed stable cubic spinel structures.

**Figure 10:**
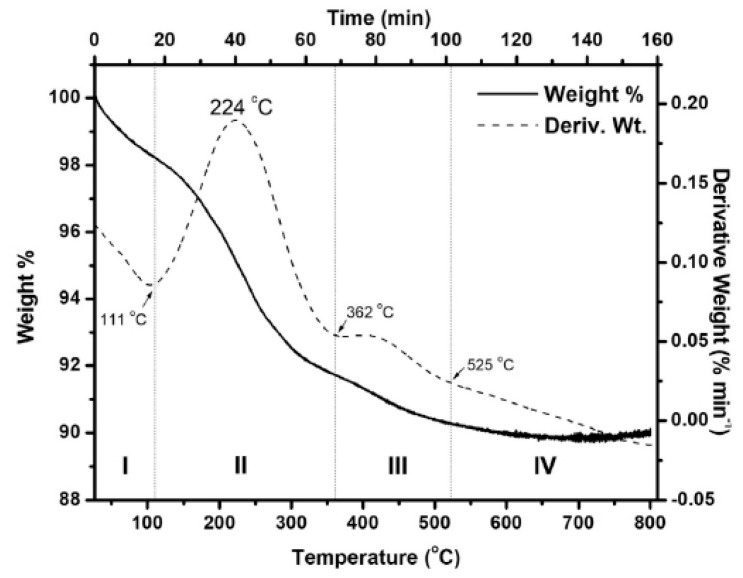
TG-DTG temperature plot of as-synthesized oxide prepared at pH ¼ 3, and f ¼ 1.0 [24], with permission from Elsevier B.V., 2014.

Thermogravimetric analysis (TGA) was employed to study the thermal stability and compositional changes of Co_3_O_4_ NPs and cobalt composite materials [24]. The TG-DTG results of the obtained Co_3_O_4_ powder are shown in Figure 10. The TG-DTG curve can be divided into several stages, where the initial pH of the precursor solution and the fuel-to-oxidizer ratio were 3.0 and 1.0, respectively. The first mass loss, observed up to 111 °C in stage I, is attributed to moisture removal and the decomposition of unreacted nitrate salts in the particles [78]. The largest weight loss occurred in stage II (6.55%), which is explained by the combined decomposition of glycine and Co(NO_3_)_2_ 6H_2_O [79,80]. During this stage, the amino groups are released, and a polymerization reaction begins, causing glycine to decompose around 215 °C [80].

In this process, flammable gases such as NH_3_, N_2_O, CO_2_, and CO are also produced [79,81]. By inhibiting the internal diffusion of oxygen, this mixture of combustible gases enhances mass transfer and heat exchange within the system and likely helps maintain the reducing environment during combustion [79]. The decomposition of the carboxyl groups in glycine may be responsible for the remaining fuel continuing to break down in stage III, resulting in an additional 1.39% weight loss [82]. At 525 °C in stage IV, no further weight loss was observed, indicating that all unreacted chemicals had been completely consumed. TGA was also used to evaluate the thermal behavior of cobalt-based composites. In the study by El-Shafie et al. (2022) [27], both OSBC and Co-OSBC samples were analyzed. No significant changes in their properties were observed within the temperature range of 100–450 °C. A weight loss of approximately 7.09% to 9.69% occurred between 50 and 100 °C, attributed to the evaporation of physically adsorbed moisture. Between 550 and 800 °C, the OSBC and Co-OSBC samples exhibited substantial weight losses of 31.06% and 38.02%, respectively, likely due to the decomposition of polymeric or other organic substances [27].

Additionally, high-resolution transmission electron microscopy (HRTEM) images reveal clear lattice planes, confirming the phase purity of Co_3_O_4_ NPs, as reported in other studies [49,83]. These nanoparticles are known for their enhanced catalytic performance, attributed to unique characteristics such as high specific surface areas (SSAs), crystallinity, and porosity [83].

The Brunauer–Emmett–Teller (BET) method was used to determine the surface area and porosity of Co_3_O_4_ NPs [84,85,86]. As shown in Table 3, variations in synthesis methods, precursor materials, and processing temperatures can significantly influence the porosity and surface area of the resulting materials. Acedera et al. (2020) [24] employed the solution combustion synthesis (SCS) method using ethylenediaminetetraacetic acid (EDTA) as the fuel to produce foam-like Co_3_O_4_ NPs with a surface area of 23 m^2^/g. The impact of fuel type and fuel-to-oxidizer ratio on the nanoparticles’ shape, internal structure, visual characteristics, and electrochemical performance was also investigated [24].

### 4.2. Application

**Figure 11:**
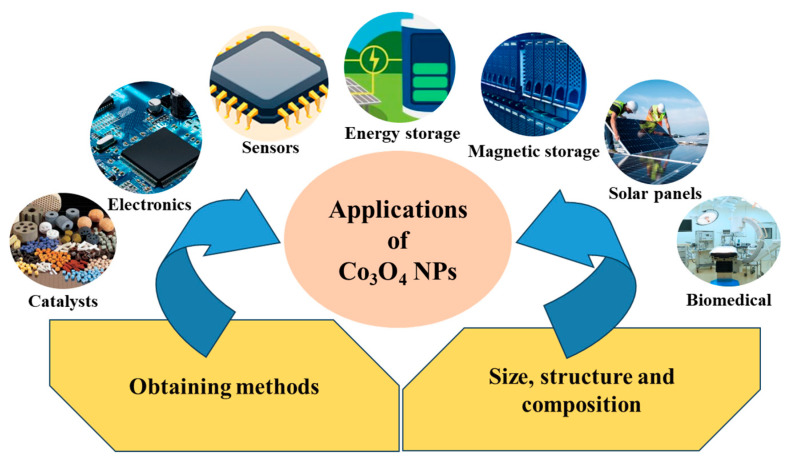
Synthesis method and morphology impact of Co_3_O_4_ NPs’ applications across industries.

Co_3_O_4_ NPs have gained considerable attention for a wide range of potential applications, including energy storage, supercapacitors, solar selective absorbing materials, anode materials in lithium-ion batteries [89], sensitive sensors, diverse catalytic processes, magneto-resistive devices, and lightweight electronic applications, as shown in Figure 11 [90,91]. These applications highlight the significance and versatility of Co_3_O_4_ NPs across various scientific and technological fields. Additionally, Co_3_O_4_ NPs demonstrate promising prospects for environmental applications such as wastewater treatment and pollutant removal, showcasing their capability to address environmental challenges [92,93]. For example, Ni-doped Co_3_O_4_ nanofibers exhibit superior performance in water splitting technology, achieving 500 mA·cm^−2^ at 1.87 V for overall water electrolysis and demonstrating an oxygen evolution reaction (OER) overpotential of 281 mV under acidic conditions [94].

The high sensitivity of Co_3_O_4_ NPs to various analytes also drives their demand in sensor applications, creating new opportunities in environmental monitoring and medical diagnostics. Farhadi et al. (2016) investigated the photocatalytic activity of Co_3_O_4_ NPs produced by thermal decomposition of a complex, revealing potential catalytic applications such as using laser-induced graphene modified with Co_3_O_4_ NPs to create a flexible, highly sensitive enzyme-free glucose biosensor [10].

In energy applications, Co_3_O_4_ NPs are promising electrode materials for lithium-ion batteries and supercapacitors. Recent research, summarized in Table 4 [37,94,95], focuses on improving their capacity performance. However, repeated cycling as an anode poses challenges due to significant volume changes. To address this, various Co_3_O_4_ nanostructures—such as nanorings, mesoporous forms, 3D nanofibers, and nanofilms—have been extensively studied [96].

#### 4.2.1. Supercapacitors and Pseudocapacitors

Co_3_O_4_ NPs demonstrate strong potential as supercapacitor and pseudocapacitor active materials because of their intrinsic pseudocapacitive characteristics, numerous redox-active sites, and their theoretical specific capacitance of ~3560 F g^−1^. The physical and chemical properties of Co_3_O_4_ NPs allow their application in supercapacitor systems using pseudocapacitor technology. Pure Co_3_O_4_ NPs show two major limitations due to their restricted electrical conductivity and moderate cycling stability. Research teams have worked to solve these limitations by improving the morphological structure and by adding heteroatoms and conductive supports. The effectiveness of these strategies to enhance electrochemical performance has been confirmed by recent studies. For instance, porous Co_3_O_4_ nanospheres produced through the SCS method demonstrated outstanding specific capacitance performance of 1323 F g^−1^ at 1 A g^−1^ current density because of their improved electroactive surface area as well as effective ion mobility through their mesoporous structure [37]. Moreover, species with oxygen vacancies generated by precision-controlled combustion synthesis reached a maximum capacity of 1186 F g^−1^ at 2 A g^−1^ due to defects enhancing redox kinetics and conductivity [97].

Doping enables significant improvements in the electrochemical performance of Co_3_O_4_-based electrodes by enhancing their essential behaviors. A modified combustion technique produced cerium-doped Co_3_O_4_ electrodes that delivered a specific capacitance of 925 F g^−1^ at 1 A g^−1^, due to Ce’s effect on the oxygen sublattice and modulation of the electronic structure [98]. Similarly, boron-doped Co_3_O_4_ NPs used in lithium-ion batteries exhibited a specific capacitance of 1096 F g^−1^ at 0.5 A g^−1^ with sustainable cycling stability exceeding 90% over 5000 cycles. This improvement is attributed to doping, which enhanced both electron mobility and the structural stability of the electrode throughout charging cycles [99]. Another highly effective approach involves combining Co_3_O_4_ with conductive carbonaceous materials like reduced graphene oxide (RGO). The RGO/Co_3_O_4_ hybrid electrode coated with chitosan achieved a specific capacitance of 1143 F g^−1^ at 0.5 A g^−1^, benefiting from the combined redox properties of Co_3_O_4_ and RGO’s high conductivity and large surface area, which enabled rapid charge transfer and improved electrolyte access [100].

Material electrochemical performance optimization strongly depends on morphological control. A low internal resistance of ~0.3 Ω, together with a capacitance of 1294 F g^−1^ at 1 A g^−1^, results from morphological characteristics that provide abundant electroactive sites and interconnected channels, enabling strong structural stability against cycling-related mechanical stress [101]. Experimental findings show that electrode interfaces perform better with hierarchical nanostructured devices that increase surface area while promoting effective ion and electron transport. The combination of MnCo_3_O_4_ derived from soot material enables asymmetric supercapacitor applications that maintain structural flexibility while achieving specific capacitance levels above 1100 F g^−1^ [102].

**Table 4:** Capacitive performance of synthesized Co_3_O_4_ NPs as electrode materials.

No.	Material	Preparation	Electrolyte	Specific Capacitance, Fg^−1^	Current Density, A g^−1^	Retention	Cycles	Ref.
1	Cobalt oxide	SCS	2 M KOH	54	10	82%	10,000	[66]
2	Cobalt oxide thin film	Heating of an alkaline bath of cobalt salt	KOH 0.25 to 2.0 M	118				[67]
3	Spinel-nanostructured Co_3_O_4_ powder	SCS		100	0.05−5	75%	100	[23]
4	Co_3_O_4_ NPs	Solid-state calcination		100	1.1		50	[4]
5	Co_3_O_4_ NPs	SCS	1 M KOH	182	0.5	71%	2000	[88]
6	Hexagonal Co_3_O_4_	SCS	6 M KOH	227	1	95%	1000	[103]
7	Co_3_O_4_ thin films	Electrodeposition	KOH	235				[94]
8	Co_3_O_4_ nanoflake	Hydrothermal	2 M KOH	351		good	4000	[104]
9	Co_3_O_4_ nanospheres	One pot hydrothermal		182	1	93.75%	8000	[90]
10	Cobalt oxide	SCS	2 M KOH	351	0.85	98.6%	1000	[95]
11	Cobalt oxide flakes	Potentiodynamic approach	0.1 M Na_2_SO_4_	396.67		better cyclic retention	1600	[105]
12	Marigold 3D flower like Co_3_O_4_ NPs	SCS	3 M KOH	603		97.6%	5000	[47]
13	Cobalt oxide	Electrode Position	PH 12	504			600	[106]
14	Co_3_O_4_@C NPs	Simple Thermolysis	2 M KOH	642	1			[38]
15	Co_3_O_4_ nanoflakes	Cathodic potential step		598.9	6.25			[107]
16	Pure Co_3_O_4_ NPs and Co_3_O_4_ /graphite nanocomposite	Co-precipitation	6 M KOH	239.5 for pure 395.04 for Co_3_O_4_/ graphite	0.5	2.68%	1000	[108]

Current knowledge demonstrates that undoped pure Co_3_O_4_ NPs have capacitance ratings ranging from 700 to 1300 F g^−1^; however, hybrid or doped systems achieve superior performance above 1500 F g^−1^, alongside enhanced lifespan and rate capabilities [109,110]. The power and energy performance of Co_3_O_4_-based electrodes in hybrid supercapacitors reach 1200 W kg^−1^ and 45 Wh kg^−1^, resulting in energy storage systems that bridge the gap with conventional lithium-ion batteries [111]. The properties of Co_3_O_4_ make these materials suitable for constructing portable energy storage platforms with flexible designs.

Beyond energy storage and supercapacitors, Co_3_O_4_ NPs exhibit significant electrochemical performance in catalytic and sensing applications due to their mixed valence states and high redox activity. These nanoparticles excel in electrochemical catalysis and sensing largely because of their ease of oxidation and reduction. Cerium-doped Co_3_O_4_ demonstrated a high specific surface area of 87.3 m^2^ g^−1^ and an exceptionally low charge transfer resistance (2.6 Ω), enhancing the efficiency of its electrochemical reactions [98]. Nitrogen-treated flower-shaped Co_3_O_4_ nanostructures displayed a specific capacitance of 1625 F g^−1^ and retained 92% of their capacity after 10,000 cycles [101]. Similarly, boron-doped Co_3_O_4_ showed improved conductivity and ion mobility, making it suitable for various advanced electrochemical devices [99]. These modifications confirm the versatility and strong potential of Co_3_O_4_ NPs in diverse electrochemical applications.

Elemental challenges remain before Co_3_O_4_ can be widely adopted for commercial supercapacitors. Nanostructured Co_3_O_4_ suffers from limited volumetric capacitance due to its dispersed packing structure, which negatively impacts practical applications. Structural degradation, electrolyte dissolution, and cycling instability further affect the long-term durability of Co_3_O_4_-based devices. Even high-performing materials often experience capacity fading after 5000 to 10,000 cycles, limiting their reliability in real-world applications.

Another major limitation is the scalability and economic feasibility of current synthesis techniques. Although combustion synthesis is attractive for its simplicity and cost-effectiveness, it often lacks precise control over particle size distribution and phase purity—both of which directly influence electrochemical performance. Moreover, the current understanding of dopant migration and its effects on crystal stability over time remains limited, despite their proven enhancement of charge storage capacity. While dopants such as Ce, B, and N have demonstrated significant performance improvements, exploring Fe, Mn, Ni, and P as dopants could open promising avenues for future advancements.

Existing research gaps require systematic investigation using operando and in situ methods to better understand the charge storage mechanisms and degradation pathways of Co_3_O_4_ electrodes. Future studies should focus on underexplored dopants, including transition metals and non-metals, through systematic screening and theoretical modeling to predict electrical interactions and analyze electrochemical responses. The advancement of environmentally friendly synthesis techniques—such as microwave-assisted combustion and plant-extract templating—must proceed in parallel to meet sustainable chemistry goals. Researchers should also seek innovative solutions to address the limitations of Co_3_O_4_ materials in electrical conductivity, cycle stability, and scalable manufacturing. Developing Co_3_O_4_-based systems for wearable and microscale energy devices will require integration with flexible substrates and solid-state configurations using novel polymer electrolytes. With ongoing breakthroughs in synthesis and material design, Co_3_O_4_ systems are poised to become core technologies in next-generation, high-performance, sustainable energy storage devices.

#### 4.2.2. Batteries

Co_3_O_4_ NPs have been successfully used as electrode materials in supercapacitors and pseudocapacitors, highlighting their potential in energy applications such as lithium-ion batteries (LIBs), lithium-ion capacitors (LICs), and emerging hybrid battery-type supercapacitors. This is due to their multivalent Co^2+^/Co^3+^/Co^4+^ redox system, high theoretical capacity (~890 mAh g^−1^), and robust structural stability. Co_3_O_4_ possesses two key properties essential for strong battery storage performance: a spinel crystalline structure and reliable electrochemical behavior. However, practical applications face challenges because charge-discharge cycling causes significant volume changes and low electrical conductivity. Current research focuses on morphological tuning and dopant incorporation to address these deficiencies. For example, Co_3_O_4_ nanostructures engineered into porous nanourchin and nanosheet morphologies improve electrolyte penetration and better accommodate strain during cycling. These morphological adjustments have been shown to enhance lithium-ion electrochemical performance, delivering capacity retention of 580–710 mAh g^−1^ over 100 cycles [112].

Doping remains a crucial strategy for overcoming these limitations and enhancing battery-type performance. As shown in Table 5, Co_3_O_4_ NPs synthesized via SCS, with or without dopants, exhibited reversible capacities ranging from 580 to 812 mAh g^−1^ and capacity retention up to 95% over 200 cycles. Incorporating cerium (Ce) into Co_3_O_4_ improves lithium intercalation kinetics and reduces charge transfer resistance by creating oxygen vacancies and enhancing structural flexibility. Ce-doped Co_3_O_4_ achieved a reversible capacity of 812 mAh g^−1^ at 0.1 A g^−1^ with 85% capacity retention after 200 cycles, outperforming pure Co_3_O_4_ [113]. Similarly, chromium (Cr) doping, along with defect creation, enhances both pseudocapacitive behavior and deep cycling capability. Cr-doped Co_3_O_4_ electrodes delivered over 720 mAh g^−1^ at 0.5 A g^−1^ with prolonged cyclability and high Coulombic efficiency exceeding 98% [114]. While doping clearly reinforces structure and improves electrochemical performance, further investigation is needed into the long-term effects of dopant migration and phase segregation during operation.

Selecting the synthesis method is also important, as it significantly affects the performance of Co_3_O_4_ NPs in potential applications. The solution combustion synthesis (SCS) method stands out as a promising technique for preparing LIB electrodes using Co_3_O_4_ NPs due to its rapid process, scalability, and precise control over particle structure. The development of nanometer-sized Co_3_O_4_ synthesized via SCS led to cells exhibiting a capacity of 630 mAh g^−1^, maintaining stable operation for eighty cycles [115]. Moreover, SCS fabrication enabled the production of uniform particles with increased surface area and enhanced porosity, delivering capacities up to 720 mAh g^−1^ over 150 cycles [23]. The development of multi-doped Co_3_O_4_ systems and their composites with other transition metal oxides holds potential for maintaining stable phase transitions while providing sustained high cycling capacities. Recent advancements in Co_3_O_4_ NP research position them as a crucial link between traditional LIBs and ultrafast pseudocapacitors, offering a flexible operational foundation for emerging hybrid energy storage technologies.

Despite significant improvements, persistent issues and research gaps remain in the development and application of Co_3_O_4_ NPs, such as capacity loss, low electronic conductivity, and unstable formation of solid electrolyte interphases (SEI), which require further attention. Scaling and reproducing the synthesis process remain challenging without standardized procedures for optimizing fuel-to-oxidizer ratios and combustion conditions in SCS. There is also a lack of long-term stability data and recyclability assessments for Co_3_O_4_-based catalysts and electrode systems under realistic operational conditions. Available studies have yet to provide satisfactory explanations regarding how structural characteristics impact specific catalytic or sensing functionalities.

Future research should focus on integrating Co_3_O_4_ with conductive carbon scaffolds, such as graphene or carbon nanotubes, while designing hierarchical porous structures to buffer mechanical stress. Additionally, comprehensive in situ observations of redox dynamics are essential. Measurement systems capable of monitoring structural modifications during chemical processes in real-time need to be developed. Expanding green synthesis methods [116], inspired by bio-based principles, will advance sustainability efforts. Synergistic advancements in sensing and energy storage can be achieved by deploying Co_3_O_4_ NPs alongside multifunctional platforms composed of carbon-based materials or metal-organic frameworks. The authors recommend further laboratory research incorporating real-world environmental factors and operational demands to bridge the gap between academic research and industrial application.

## 5. Conclusions

The unique features of Co_3_O_4_ NPs—such as their redox-active surfaces, spinel structure, narrow band gap, and excellent thermal and chemical stability—make them a leading multifunctional material of the current decade. This review highlights the synthesis of Co_3_O_4_ NPs via the SCS method, which offers a rapid and cost-effective approach to producing morphologically diverse, porous nanostructures.

The performance and properties of materials synthesized by SCS are strongly influenced by three key parameters: fuel type, fuel-to-oxidizer ratio (φ), and reaction temperature. Adjusting φ from 0.5 to 2.0 yields nanoparticles ranging from 12 to 70 nm and produces three distinct generations of specific capacitance, spanning 362.8 to 3560 F·g^−1^, depending on fuel choice and calcination conditions.

Co_3_O_4_ NPs show notable promise in energy applications, achieving capacities exceeding 1000 mAh·g^−1^ in lithium-ion batteries and offering high specific capacitance with rapid charge–discharge in supercapacitors. Additionally, doping with Ni and Ce significantly enhances their catalytic activity for oxygen evolution reactions (OER) and CO_2_ reduction.

Despite these advantages, practical implementation faces challenges such as long-term stability, scalability within green chemistry frameworks, and integration into hybrid multifunctional systems. Addressing these issues could involve coating Co_3_O_4_ NPs with carbon or other conductive materials to reduce structural defects and improve durability. Moreover, integrating Co_3_O_4_ NPs with biosensing platforms presents opportunities for multifunctional applications. These strategies underscore the importance of tailored nanostructures and sustainable production methods to enhance performance across a wide range of potential uses

## Figures and Tables

**Table 1:** The different process conditions and parameters in the SCS synthesis of Co_3_O_4_ nanostructures.

No.	Fuel	F/O Ratio(φ)	Temperature (°C)	Particle Size nm	Morphology	Ref.
1	Glycine	1	500	20	Agglomerated NPs	[23]
2	Urea	0.75–1.25	400–500	30–70	Porous clusters	[19]
3	Citric acid	1.0–2.0	300–600	25–50	Crystalline porous	[19]
4	Glycine	1.2	500	45	Hollow spheres	[37]
5	Plant extract	1.5	450	<50	Spherical porous	[38]

**Table 3:** A comparison of the BET analysis results for specific surface area (SSA) corresponding to the preparation temperatures of several Co_3_O_4_ nanostructures.

No.	Synthesis Method	Raw Materials	Catalyst	Temperature, °C	SSA, m^2^g^−1^	References
1	SCS	(C_4_H_6_O_4_Co·4H_2_O) and d-(+)(C_6_H_12_O_6_)	Spinel-structured Co_3_O_4_ powder	700	3	[23]
2	SCS	(Co(NO_3_)_2_·6H_2_O and urea ((NH_2_)_2_CO)	Co_3_O_4_ NPs	300–800	39–2	[63]
3	Hydrothermal	(Co(NO_3_)_2_·6H_2_O) and urea (CO(NH_2_)_2_) in deionized water	Co_3_O_4_ nanoplate, nanorod, NPs	325	45.5–111.4, 112.6	[87]
4	Sol–gel	(Co(NO_3_)_2_·6H_2_O) and (C_2_H_5_-OH)	Co_3_O_4_ NPs	150–550–650	15–46–42	[84]
5	Sol–gel	(Co(NO_3_)_2_·6H_2_O) and PEG in deionized water	Co_3_O_4_ nanorod	90–350–700	170.2–48–20.9	[85]
6	Reactive calcination route	(Co(NO_3_)_2_·6H_2_O) and (Mn(NO_3_)_2_·4H_2_O) in deionized water	Mn promoted Co_3_O_4_ spinel (Cat-R)	340–380–420	127.94–94.5–57.43	[88]
7	SCS	(Co(NO_3_)_2_·6H_2_O) and urea (CO(NH_2_)_2_) in deionized water	Nano-crystalline Co_3_O_4_	600	10	[67]
8	Co-precipitation	(CoCl_2_. 6H_2_O), (AgNO_3_) and (NH_3_), in deionized water	Single-cubic Co_3_O_4_ nanostructure Ag doped		407.33	[11]

**Table 5:** Comparative electrochemical performance of Co_3_O_4_ NPs synthesized by the SCS method for battery applications.

No.	Synthesis Method	Dopant	Specific Capacity (mAh g^−1^)	Cycling Stability (Retention% @ Cycles)	Ref.
1	SCS (morphology-controlled)	None	580–710	90 @ 100	[112]
2	SCS	Ce	812	85 @ 200	[113]
3	SCS	Cr	>720	95 @ 200	[114]
4	SCS	None	630	80 @ 80	[115]
5	SCS (refined)	None	>720	90 @ 150	[23]
6	Advanced SCS + conductive composite	Multi-doping (e.g., Ce + Cr)	>700	Needs investigation (≥300 cycles desired)	Future direction
